# C–H Functionalization

**DOI:** 10.3762/bjoc.8.176

**Published:** 2012-09-18

**Authors:** Huw M L Davies

**Affiliations:** 1 Department of Chemistry, Emory University, 440 Atwood Hall, 1515 Dickey Drive, Atlanta GA 30322, USA

**Keywords:** C–H Functionalization

C–H Functionalization has the potential to become a paradigm-shifting strategy for organic synthesis. Over the last decade, the field has experienced explosive growth and a large variety of new C–H functionalization methodologies have been developed. In particular, regioselective functionalization of sp^2^ C–H bonds has become a broadly flexible approach for the synthesis of complex aromatic carbocycles and heterocycles. In several instances the substrates have a natural preference for functionalization at specific C–H bonds. Alternatively, selective functionalization is achieved by using a directing group to orient the catalyst in a defined position. These types of synthetic strategies are already having a significant impact on the streamlined synthesis of important compounds for the pharmaceutical industry and materials science.

The selective functionalization of sp^3^ C–H bonds is a more challenging proposition, but in recent years significant advances have been made to suggest that even these types of transformations can become broadly applicable. Metal-bound carbenes, nitrenes and oxo species have been particularly effective at stereoselective sp^3^ C–H functionalization. However, considerable advances still need to be made to enhance the selectivity and to increase the range of functionality that can be introduced in these types of reactions.

The fundamental principles of a number of C–H functionalization transformations have been established but, in many regards, the field is still in its infancy. The ultimate goal would be to have generally programmable and controllable methods for the highly selective C–H functionalization of complex systems at will. To achieve this, it will be necessary to have an extensive toolbox of catalysts and reagents to override the natural site selectivity of any given substrate. Therefore, a greater range of reaction types need to be developed and a better mechanistic understanding of the controlling elements of the various methods has to be obtained.

This Thematic Series highlights some of the novel approaches that are applied to the field of C–H functionalization and I thank all the authors for their exciting contributions. The series covers topics that range from novel catalyst design, new synthetic methods, and cascade sequences that incorporate C–H functionalization. The articles illustrate the exciting opportunities for innovation that exist in C–H functionalization research, and hopefully, will inspire others to explore new research directions in this area.

Huw M. L. Davies

Atlanta, September 2012

